# The Effect of Medial Tibial Slope on Anterior Tibial Translation and Short-Term ACL Reconstruction Outcome

**DOI:** 10.1055/s-0038-1669929

**Published:** 2018-09-10

**Authors:** Steffen Sauer, Mark Clatworthy

**Affiliations:** 1 Department of Sports Traumatology, Aarhus University Hospital, Aarhus, Denmark; 2 Department of Orthopaedic Surgery, Middlemore Hospital, Auckland, New Zealand

**Keywords:** ACL, slope, ACL failure

## Abstract

**Background** Increased tibial slope has been shown to be associated with higher anterior cruciate ligament (ACL) reconstruction failure rate. Little is known about the correlation of tibial slope and anterior tibial translation in ACL deficient and reconstructed knees as well as the correlation of tibial slope and ACL reconstruction outcome.

**Purpose/Hypothesis** The purpose of this study was to investigate the correlation of tibial slope with anterior tibial translation and ACL reconstruction outcome. It is hypothesized that increased medial tibial slope is associated with increased anterior tibial translation in the ACL deficient knee. Medial tibial slope is neither expected to affect anterior tibial translation in the ACL reconstructed knee nor short-term ACL reconstruction outcome.

**Materials and Methods** A cohort of 104 patients with unilateral isolated ACL deficiency undergoing hamstring ACL reconstruction by a single surgeon between 2002 and 2004 was followed up prospectively. Preoperative data were collected including patient demographics, time to surgery, subjective and objective International Knee Documentation Committee (IKDC) outcome scores, as well as manual maximum anterior tibial translation measured with the KT-1000 measuring instrument. Medial tibial slope was assessed on long lateral X-rays using the method described by Dejour and Bonnin (1994). Intraoperative data were collected including meniscal integrity; postoperative data were collected at 1-year follow-up including manual maximum anterior tibial translation (KT-1000 measured), and subjective and objective IKDC scores.

**Results** A significant positive correlation was seen between medial tibial slope in ACL deficient knees and KT-1000–measured anterior tibial translation ( *r* = 0.24; *p* = 0.003). The positive relationship increased when meniscal integrity was factored in ( *r* = 0.33; *p* < 0.001). No significant correlation was seen between medial or lateral meniscal integrity and KT-1000–measured anterior tibial translation ( *r* = −18; *p* = 0.06). No significant correlation was seen between KT-1000–measured anterior tibial translation and time to surgery. One year postoperatively, 82 patients were assessed, while 26 patients were lost to follow-up; no significant correlation was found between increased medial tibial slope and poor ACL reconstruction outcome measured by post-ACL reconstruction anterior tibial translation (KT-1000) or subjective and objective IKDC scores.

**Conclusion** Increased medial tibial slope is associated with increased (KT-1000 measured) anterior tibial translation in ACL deficient knees. No significant correlation is found between increased medial tibial slope and poor short-term ACL reconstruction outcome.

Rupture of the anterior cruciate ligament (ACL) represents a common knee injury, eventually resulting in long-term complications such as meniscal and chondral deterioration even after the ACL has been satisfactorily reconstructed and stable conditions are restored. 1 2 Thus, numerous studies have focused on possible risk factors favoring ACL injuries to reduce the incidence of ACL injuries and ACL reconstruction failures. 3 4 5 6 In addition to young patient age, high level of function as well as physiological factors such as neuromuscular control, the geometrical shape of the knee joint including the posterior tibial slope has been identified to influence the susceptibility of ACL injuries and ACL graft failures. 7 8 9 10 11 12 13 14 15 Increased anterior tibial translation is thought to be the primary mechanism for this finding. However, the influential strength of these geometrical factors remains unknown and may be confounding; factors which influence the susceptibility of ACL injuries and graft failures are mutually dependent including the incidence of meniscal injuries, patient age, and tibial slope. The purpose of this prospective study was to determine the effect of medial tibial slope on anterior tibial translation in the ACL deficient and reconstructed knee using the KT-1000 measuring instrument and to investigate the effect of medial tibial slope on meniscal integrity and ACL reconstruction outcome. It is hypothesized that increased medial tibial slope is associated with increased anterior tibial translation in the ACL deficient knee. Medial tibial slope is not expected to affect anterior tibial translation in the ACL reconstructed knee as the translation is limited by the reconstructed ACL graft. Furthermore, medial slope is not expected to affect short-term ACL reconstruction outcome; however, increased medial tibial slope may increase graft strain 13 16 and long-term follow-up may eventually indicate that increased medial tibial slope is associated with higher ACL reconstruction failure rate.

## Materials and Methods

A cohort of 104 patients with unilateral isolated ACL deficiency undergoing hamstring ACL reconstruction by a single surgeon between 2002 and 2004 was followed up prospectively. Preoperative data were collected including patient demographics, time to surgery, subjective and objective IKDC outcome scores, as well as manual maximum anterior tibial translation measured with the KT-1000 measuring instrument. Furthermore, medial tibial slope was assessed on long lateral X-rays using the method described by Dejour and Bonnin which has shown acceptable inter- and intraobserver reliability. 17 18 The medial posterior tibial slope was hereby measured as the angle formed by the tangent to the medial tibial plateau and perpendicular to the lateral mechanical axis through the middle of the medial tibial plateau and the center of the talus. Intraoperative data were collected including meniscal integrity and percentage of meniscal tissue remaining after partial meniscectomy when performed. A transtibial ACL reconstruction technique was used in all cases, femoral fixation was ensured with the Endobutton CL, while tibial fixation was ensured with the Intrafix interference screw. All patients underwent the same accelerated rehabilitation program. Postoperative data were collected at 1-year follow-up including manual maximum anterior tibial translation (KT-1000 measured), and subjective and objective IKDC scores. Data were assessed and analyzed for significant correlation using regression analysis and *t* -tests.

## Results

In this study, 73 males and 31 females (mean age: 29 years) with unilateral isolated ACL deficiency were assessed; ACL injury was sustained sports related in 73 cases. Preoperative objective IKDC score was severely abnormal in 48 patients and abnormal in 56 patients. None of the patients showed a nearly normal or normal IKDC score. Mean preoperative subjective IKDC score was 54 (range: 24–79). Mean tibial slope in ACL deficient knee was 8.36 degrees (range: 0–17 degrees). Mean anterior tibial translation of ACL deficient knees measured by KT-1000 was 6.38 mm (range: 3–19 mm). A significant positive correlation was seen between medial tibial slope of ACL deficient knees and the KT-1000–measured anterior tibial translation ( *r* = 0.24; *p* = 0.003). Meniscal integrity and meniscal treatment procedure (in per cent) are shown in Table 1 . Partial medial meniscectomy was performed in 24% of cases, while lateral partial meniscectomy was performed in 8% of cases. Meniscal repair was performed in 19% of cases, while lateral meniscal repair was performed in 8% of cases. No significant correlation was seen between medial or lateral meniscal integrity and KT-1000–measured anterior tibial translation ( *r* = −18; *p* = 0.06). No significant correlation was seen between KT-1000–measured anterior tibial translation and time to surgery. One year postoperatively, 82 patients were assessed, while 22 patients were lost to follow-up; 93% of patients showed a post-ACL reconstruction anterior tibial translation (KT-1000 measured) of less than 3 mm. One patient showed a post-ACL reconstruction anterior tibial translation (KT-1000 measured) of more than 5 mm and was defined as an ACL failure. Mean postoperative subjective IKDC score was 90 (63–100). Seventy-four patients showed a normal objective IKDC score, three patients showed a nearly normal score, while one patient showed a severely abnormal objective IKDC score.

**Table 1:** Meniscal integrity and performed meniscal treatment procedure in per cent

	Medial meniscus	Lateral meniscus
Normal	52%	66%
Partial meniscectomy	24%	18%
Meniscal repair	19%	8%
Stable tear—no surgery	5%	9%

## Discussion

The primary finding of this study was that increased medial tibial slope is associated with increased (KT-1000 measured) anterior tibial translation in ACL deficient knees. Second, no significant correlation was found between increased medial tibial slope and poor short-term ACL reconstruction outcome at 1-year follow-up measured by post-ACL reconstruction anterior tibial translation (KT-1000 measured) as well as subjective and objective IKDC scores. To date (March 2017), three patients with ACL reconstruction failure have been identified with tibial slopes of 10, 11, and 17 degrees, respectively, which all lie above the average measured tibial slope of 8.36 degrees. The findings of this study support the findings of Dejour and Bonnin, who demonstrated a mean 6 mm increase in anterior tibial translation for each 10 degrees increase in posterior tibial slope in ACL deficient knees. 17 Furthermore, numerous studies have shown a correlation between increased tibial slope and susceptibility of ACL injuries. 8 9 19 20 21 22 However, there is no consensus regarding a specific tibial cutoff slope that is related to a particularly high risk of ACL injury. In addition, the predictive strength of medial and lateral tibial slopes may be dependent on the accuracy of the slope measuring method. 21 23 A systematic review by Wordeman et al showed that medial slope measurements preformed on lateral X-rays were more likely to show significant slope differences in ACL deficient knees versus control groups compared with medial slopes measured on magnetic resonance imaging (MRI). However, a majority of MRI-based slope measurement studies have identified the lateral tibial slope to be a stronger predictor of the susceptibility of ACL injuries. 21 Little is known about the correlation of tibial slope and anterior tibial translation in ACL deficient and reconstructed knees. Li et al investigated 40 patients retrospectively who had undergone ACL reconstruction and found a significant positive correlation between medial and lateral tibial slopes (measured on MRI) and anterior tibial translation (measured by KT-1000) in ACL reconstructed knees at 2-year follow-up. 24 In our study, no significant correlation was found between medial slope and post-ACL reconstruction anterior tibial translation (measured by KT-1000) at 1 year follow-up; it is conceivable that the association of increased tibial slope and increased anterior tibial translation may become more evident over time. An increasing body of literature is indicating that the susceptibility of ACL injuries is influenced by different factors including young patient age, graft size, graft type, high level of function, neuromuscular control, and the geometrical shape of the knee joint including the intercondylar notch and the posterior tibial slope. 4 5 6 12 14 25 26 However, the mutual dependency of some of these factors may be confounding, for example, numerous studies have shown significant higher ACL reconstruction failures in young patients which comprise several factors which increase ACL injury susceptibility such as smaller graft size, higher level of function, higher incidence of meniscal injuries, and lower compliance to rehabilitation protocols. Increased susceptibility of ACL injuries and ACL graft failures is multifactorial and increased tibial slope should be considered one of these factors. If lateral or medial tibial slope is estimated to be increased when assessed on MRI, a long lateral X-ray of the lower limb should be considered to re-evaluate medial and lateral slopes. Further studies with longer follow-up including the measurement of anterior tibial slope are needed in the future. The strength of this study is that tibial slope measurements have been performed on long lateral X-rays which have been shown to have a high sensibility for correlation between slope and anterior tibial translation. The weakness of this study is that only medial slopes have been measured. A re-evaluation of lateral slopes is not feasible as X-rays are no longer available due to the long follow-up. Furthermore, we have chosen a 1-year follow-up which may be too early to depict the association of tibial slope and poor outcome including anterior tibial translation.

## Conclusion

Increased medial tibial slope is associated with increased (KT-1000 measured) anterior tibial translation in ACL deficient knees. No significant correlation is found between increased medial tibial slope and poor ACL reconstruction outcome.
